# Malaria frontline project: pre-intervention Malaria baseline assessment in Kano and Zamfara States, August 2016

**DOI:** 10.11604/pamj.supp.2021.40.1.18809

**Published:** 2021-11-12

**Authors:** Adefisoye Oluwaseun Adewole, Shelby Cash, Amina Umar, Olufemi Ajumobi, Usaini Bala, Ndadilnasiya Waziri, Saheed Gidado, Ieren Isaac, Gideon Ugbenyo, Edwin Simple, Patrick Nguku, Anisa Saleh, Steve Yoon, Stephen Patrick Kachur, Kwame Asamoa

**Affiliations:** 1 African Field Epidemiology Network, Abuja, Nigeria,; 2 Malaria Branch, Division of Parasitic Diseases and Malaria, Center for Global Health, US Centers for Disease Control and Prevention, Atlanta, Georgia,; 3 Mailman School of Public Health, Columbia University, New York, New York

**Keywords:** Malaria, assessment, diagnosis, surveillance, LLIN, Nigeria

## Abstract

**Introduction:**

In 2016, the Centers for Disease Control and Prevention and the Government of Nigeria initiated the Malaria Frontline Project in Kano and Zamfara States. The project goal is to improve the quality and coverages of malaria interventions adapting polio program strategy. We conducted a baseline assessment of malaria interventions.

**Methods:**

Twenty-four primary health centers per State were selected using probability sampling. Health workers (HW) were purposively sampled to assess their knowledge of national malaria control guidelines. Clients were selected for exit interview to assess health workers´ adherence to the national guidelines. WHO cluster methodology was used to survey heads of household and women of reproductive age on knowledge of malaria prevention, Long Lasting Insecticidal Net (LLIN) ownership and use.

**Results:**

Of the 158 HW interviewed, 94.3% knew the correct criteria for malaria diagnosis, 86.1% reported using artemisinin-based therapy to treat uncomplicated malaria. About 45% of HW reported prescribing artemisinin-based combination therapy (ACT) for uncomplicated malaria in first trimester of pregnancy and 39% prescribed quinine. Only 73.9% of fever cases were referred to laboratory as recommended by the national guideline. Households with one LLIN per 2 persons (Kano: 27.1%; Zamfara: 30.0%), LLIN use (Kano: 70.8%; Zamfara: 81.6%) and IPTp1 (Kano: 38.6%; Zamfara: 33.3%).

**Conclusion:**

most clinicians have knowledge of national guidelines, but fewer adhere to guidelines in practice. Population LLIN ownership, LLIN use among pregnant women and IPTp are lower than the national targets of 58%, 83% and 75% respectively for 2016. We recommend improving health workers´ technical capacity and adherence to national malaria guidelines.

## Introduction

Globally, an estimated 216 million cases of malaria occurred in 2016 with 445,000 deaths. Nigeria contributed 27% of the cases, the largest proportion of any country [1]. Despite progress made in the past decade to scale up malaria control interventions, malaria burden in Nigeria remains unacceptably high. To leverage the substantial human and technical capacity within the National Stop Transmission of Polio Program (NSTOP) and Nigeria Field Epidemiology and Laboratory Training Program (NFELTP), the Malaria Frontline Project (MFP), a collaborative 3-year initiative to support the Nigeria National Malaria Elimination Program (NMEP), was launched with support from the US Centers for Disease Control and Prevention (CDC). The MFP´s goal is to improve the quality of implementation of WHO recommended malaria interventions being supported by malaria partners. The objectives of the project are to: 1) strengthen technical capacity of health workers to implement quality malaria intervention at the local government area (LGA). 2) Improve the quality of malaria surveillance and facilitate evidence-based decision making to increase Nigeria´s public health capacity to prevent, detect, and respond to epidemics and other endemic high-impact diseases. The MFP builds on the experiences of NSTOP from the polio eradication and Ebola responses to strengthen health workers´ capacity to analyze and use malaria surveillance data for decision-making [2-5]. The MFP is collaborating at the field level with other malaria stakeholders including the US President´s Malaria Initiative (PMI), UK Department of International Development (DFID), WHO and UNICEF [5-7]. Kano and Zamfara States were selected for the MFP implementation because of high political commitment from State authorities, high malaria burden but scarce resources related to technical capacity. The MFP has technical staff at national, State, and LGA levels who support the technical work of malaria program staff and other HWs. The MFP staff at LGA are known as National Stop Transmission of Polio Local Government Officers (NSLOs). The MFP uses the Comprehensive Quality Improvement method (CQI), so staff meet regularly to analyze identified problems, find appropriate solutions and modify implementation strategy accordingly. The MFP has adapted NSTOP´s polio eradication thematic training approach of didactic classroom teaching followed by field assignments. The NSLOs join the malaria team at the LGA level for on-the-job mentoring during health facility supportive supervisory visits. Identified problems from these field visits are noted for team discussions and solution at monthly team meetings. A cross-sectional malaria baseline assessment was conducted in August 2016 to assess the status of Malaria control implementation in Zamfara and Kano States just before commencement of the project. The team assessed knowledge and practices of HWs on malaria diagnosis and treatment, community knowledge of malaria interventions, intermittent prophylactic treatment in pregnancy (IPTp) coverage, Long-lasting insecticidal net (LLIN) ownership and use. Results from the assessment informed the contents of the manuals prepared for the thematic trainings of staff to improve program implementation. The assessment will also serve as the baseline to measure the effect of the project on key malaria indicators at the end of the project.

## Methods

### Study area

**Figure 1:**
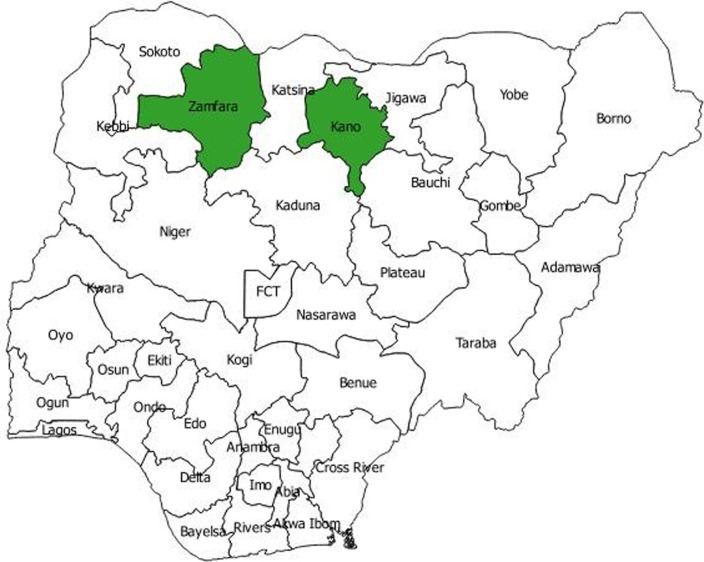
Map of Nigeria showing Kano and Zamfara States

The study areas are in Kano and Zamfara States, north-west geo-political zone of Nigeria (Figure 1). At the time of the baseline assessment, Kano State had a population of 12,945,338 (projected from 2006 census) with over 1,000 Primary Health Care units (PHCs), 36 general hospitals (GHs) and 2 tertiary facilities. Zamfara had a population of 4,466,775 (projected from 2006 census) with nearly 700 PHCs, 19 GHs and 1 tertiary facility. Both States have a cadre of public health professionals who are graduates of the NFELTP and now employed by NSTOP. These public health professionals are supporting polio eradication and helping to improve routine immunization services. From the 2015 National Malaria Indicator Survey (NMIS), the prevalence of malaria parasitemia was 60.2% and 69.9% in Kano and Zamfara, respectively, by malaria rapid diagnostic test (RDT) among eligible children of 6-59 months. The percentage of households with at least one LLIN was 88% and 89%, in Kano and Zamfara States, and LLIN use the night before survey was 43.8% in Kano and 56.3% in Zamfara [8]. MFP is implemented in every health facility of all the 14 LGAs of Zamfara State and every health facility in 20 of the 44 LGAs in Kano State. The restriction to 20 LGAs in Kano State was due to availability of project funds. The malaria program is supported by PMI and other partners in Zamfara State and by DFID and other stakeholders in Kano.

This was a cross-sectional study of health workers, care seekers and community members. The health worker assessment involved key informant interviews and an assessment of knowledge and practices in malaria diagnosis and treatment. Care seekers from Outpatient Department (OPD) were interviewed after receiving medical services and exiting from the primary health center (PHC) to ascertain HWs adherence to the national malaria guidelines of testing all febrile cases for malaria. Client exit interviews were conducted at PHCs but not at general hospitals (GHs). For community members, the heads of household and women of reproductive age (15-49 years) in the household were interviewed on knowledge on malaria prevention, Intermittent Preventive Treatment (IPTp), LLIN ownership and use.

### Sampling

For the health facility assessment, probability based on patient load was used to select 24 PHCs in each State. Though 24 PHCs were selected per State, 23 facilities were actually surveyed in each State. One PHC was inaccessible in each State due to security reasons and the inaccessible facilities were not replaced. The general hospital serving as referral facility for the selected PHCs were also included in the survey. In each health facility, the staff attending to patients and the facility head were interviewed (158 HWs). A maximum of five individuals seeking services at the OPD because of fever on the survey day were consecutively and purposively selected for exit interview to ascertain HWs adherence to the national guidelines on malaria diagnosis. One hundred and thirty-one patients with fever visiting OPD participated in the client exit interview.

The household survey was conducted using the WHO cluster survey methodology; 30 computer generated enumeration areas (EAs) were selected for each State. The EAs are clusters of households arranged by population size in descending order; 30 were selected using determined sampling interval based on number of EAs. A cluster is a primary sampling unit serving as base for census enumeration. The National Population Commission has a list of 20,249 clusters in 20 LGAs in Kano State and 17,449 cluster in the 14 LGAs in Zamfara State. The cluster sampling was followed by selecting seven households (HHs) in each cluster. A starting point within each cluster was identified using Orux-mapping through android mobile phone with GPS technology. Facing north, the first HH was identified and subsequent selections were made by making a right turn until seven eligible HHs were interviewed. Overall, 210 households (7 households from 30 clusters) were surveyed in each State.

### Data collection

Fourteen survey teams were formed for Zamfara and 20 teams were formed for Kano (one team for each LGA). The survey team consisted of personnel from the NSTOP, NFELTP residents, NMEP and Ministry of Agriculture. A survey team was made up of 3-4 data collectors (NFELTP residents) and one supervisor who was an NFELTP graduate. The teams were trained on how to access and administer the questionnaires and enter data using the Open Data Kit (ODK), an android-phone base data collection program. At each health facility, two HWs (head of facility and a HW who attends to patients) were interviewed using a structured questionnaire for their knowledge and practice of malaria case management. A questionnaire was also used for the client exit interview.

In the community, the household head was interviewed for household net coverage and eligible women of reproductive age were interviewed for their knowledge on malaria illness, treatment, IPTp, LLIN ownership and use. Women were interviewed with the assistance of a female interpreter fluent in both English and the local language, Hausa. A community member engaged by the survey team introduced the team to the household and was present during the interview to conform with the cultural norms of the study population.

### Data quality control, processing and analysis

MFP staff coordinated the survey. NFELTP graduates with at least three years´ experience in public health supervised the assessment team. Team supervisors cross-checked data collected at the end of each day before the data management team upload the cleared data on web-based platform. Data entry, cleaning and analysis were conducted using SPSS Software version 20. Descriptive statistics were conducted on categorical datasets. The questionnaires allowed multiple answers to a question. The results were not adjusted for sampling.

### Ethical consideration

Ethical approval for the assessment was obtained from Kano and Zamfara State Institutional Review Boards; the survey was approved as a non-research activity by the Office of the Associate Director for Science, Center for Global Health at CDC. Oral informed consent was obtained from HWs while written consent was obtained from household survey respondents and participants of client exit interviews.

## Results

One hundred and fifty-eight HWs (Kano: 83, Zamfara: 75), of which 81 (51%) work in PHCs (Kano: 38, Zamfara: 43) and 77 (49%) work in GHs (Kano: 45, Zamfara: 32) were interviewed.

### Health workers´ knowledge and practices on malaria treatment

One hundred and forty-nine (94.3%) of the respondents had “correct” knowledge of the diagnostic criteria (clinical symptoms plus parasitological confirmation) for malaria. Of the HWs interviewed, 136 (86.1%) reported prescribing artemisinin-based combination therapy (ACT), the NMEP recommended first-line treatment for uncomplicated malaria. Small proportions mentioned using chloroquine (6.3%), and antibiotics (5.7%) for malaria case management, although these are not recommended drugs for malaria treatment by NMEP (Table 1, Table 2).

**Table 1:** knowledge and practice on malaria case management among health workers interviewed states

Characteristics		Kano N=83% (95% CI)	Zamfara N=75% (95% CI)	Total N=158 (%)
**Knowledge on diagnostic criteria for Malaria**	Clinical diagnosis	2.4 (-0.009-0.057)	4.0 (-0.004-0.084)	3.2
	Clinical plus parasitological diagnosis	93.9 (0.888-0.990)	94.7 (0.896-0.998)	94.3
	No response	3.6 (-0.004-0.076)	1.3 (-0.012-0.039)	2.5
**Reported treatment provided for uncomplicated malaria**	Artemisinin-based combination therapy	85.5 (0.779-0.931)	86.7 (0.790-0.944)	86.1
	Artesunate monotherapy	19.3 (0.108-0.278)	25.3 (0.155-0.351)	22.2
	Sulphadoxine pyrimethamine	19.3 (0.108-0.278)	20.0 (0.109-0.291)	19.6
	Quinine	33.7 (0.235-0.439)	22.7 (0.132-0.322)	28.5
	Chloroquine	8.4 (0.024-0.144)	4.0 (-0.004-0.084)	6.3
	Antibiotics	7.2 (0.016-0.128)	4.0 (-0.004-0.084)	5.7

* multiple response

**Table 2:** knowledge and practice on malaria case management among health workers interviewed states (facility type)

Characteristics		Kano		Zamfara	
		PHCs N=38 % (95% CI)	GHs N=45 % (95% CI)	PHCs N=43 % (95% CI)	GHs N=32 % (95% CI)
**Knowledge on diagnostic criteria for Malaria**	Clinical diagnosis	0.0	4.4 (-0.016-0.104)	4.7 (-0.016-0.110)	3.1 (-0.029-0.091)
	Clinical plus parasitological diagnosis	97.4 (0.923-1.025)	91.1 (0.828-0.994)	95.3 (0.890-1.016)	93.8 (0.854-1.022)
	No response	2.6 (-0.024-0.077)	4.4 (-0.016-0.104)	0.0	3.1 (-0.029-0.091)
**Reported treatment provided for uncomplicated malaria**	Artemisinin combination therapy	84.2 (0.726-0.958)	86.7 (0.768-0.966)	86.0 (0.756-0.964)	87.5 (0.760-0.990)
	Artesunate monotherapy	13.2 (0.024-0.240)	24.4 (0.119-0.369)	18.6 (0.070-0.302)	21.9 (0.075-0.362)
	Sulphadoxine pyrimethamine	21.1 (0.081-0.341)	17.8 (0.066-0.290)	25.6 (0.126-0.386)	25.0 (0.010-0.400)
	Quinine	34.2 (0.191-0.493)	33.3 (0.195-0.471)	18.6 (0.070-0.302)	28.1 (0.125-0.437)
	Chloroquine	2.6 (-0.025-0.066)	13.3 (0.033-0.232)	7.0 (-0.006-0.146)	0.0
	Antibiotics	7.9 (-0.007-0.165)	6.7 (-0.006-0.140)	4.7 (-0.016-0.110)	6.3 (-0.021-0.147)

* multiple response

### Health workers practices on treatment of Malaria in pregnancy

Of surveyed HWs, 39.9% reported treating uncomplicated malaria in the first trimester of pregnancy with quinine, 45.6% prescribed ACT and 20.3% prescribed sulphadoxine-pyrimethamine (SP). In the second and third trimester of pregnancy, ACT was the most commonly used drug for uncomplicated malaria treatment (80.4%) followed by quinine (19.0%). The national guidelines recommend quinine during the first trimester and ACT in the second and third trimester (Table 3, Table 4).

**Table 3:** reported treatment of malaria illness during pregnancy among health workers interviewed

		Kano	Zamfara	Total
Health worker practices		N=83 % (95% CI)	N=75 % (95% CI)	N=158 (%)
**Drugs given for uncomplicated malaria in first trimester of pregnancy**	Chloroquine	4.8 (0.002-0.093)	1.3 (-0.012-0.039)	3.2
	Quinine	43.4 (0.327-0.541)	36.0 (0.251-0.469)	39.9
	Sulphadoxine pyrimethamine	20.5 (0.118-0.292)	20.0 (0.109-0.291)	20.3
	Artemisinin-based combination therapy	38.6 (0.281-0.491)	53.3 (0.420-0.646)	45.6
**Drugs given for uncomplicated malaria in 2nd and 3rd trimesters**	Quinine	25.3 (0.159-0.347)	12.0 (0.046-0.194)	19.0
	Sulphadoxine pyrimethamine	19.3 (0.108-0.278)	9.3 (0.027-0.159)	14.6
	Artemisinin-based combination therapy	72.3 (0.049-0.819)	89.3 (0.823-0.963)	80.4

* multiple response

**Table 4:** reported treatment of malaria illness during pregnancy among health workers interviewed (facility type)

Health worker practices		Kano		Zamfara	
		PHCs N=38 % (95% CI)	GHs N=45 % (95% CI)	PHCs N=43 % (95% CI)	GHs N=32 % (95% CI)
**Drugs given for uncomplicated malaria in first trimester of pregnancy**	Chloroquine	7.9 (-0.007-0.165)	2.2 (-0.021-0.065)	2.3 (-0.022-0.068)	0.0
	Quinine	47.4 (0.315-0.633)	40.0 (0.257-0.543)	34.9 (0.207-0.491)	37.5 (0.207-0.543)
	Sulphadoxine pyrimethamine	23.7 (0.102-0.372)	17.8 (0.066-0.290)	20.9 (0.087-0.331)	18.8 (0.053-0.323)
	Artemisinin-based combination therapy	21.1 (0.081-0.341)	53.3 (0.387-0.679)	60.5 (0.459-0.751)	43.8 (0.266-0.610)
**Drugs given for uncomplicated malaria in 2nd and 3rd trimesters**	Quinine	28.9 (0.145-0.433)	22.2 (0.101-0.343)	18.6 (0.070-0.302)	6.3 (-0.021-0.147)
	Sulphadoxine pyrimethamine	23.7 (0.102-0.372)	17.8 (0.066-0.290)	9.3 (0.006-0.180)	9.4 (-0.007-0.195)
	Artemisinin-based combination therapy	63.2 (0.479-0.785)	80.0 (0.683-0.917)	86.0 (0.756-0.964)	93.8 (0.854-1.022)

* multiple response

#### Client exit interview

A total of one hundred and thirty-one clients with fever were interviewed in the 46 PHCs. Of these, 74.5% were referred by HW for laboratory confirmation of malaria diagnosis as recommended by the national guidelines. Over 80% received their laboratory result in less than 1 hour and almost 90% of all laboratory referred patients returned with their results to the HW. However, only 76.1% received explanation of their results from the HW (Table 5).

**Table 5:** results of client interview

	Kano State (%)	Zamfara State (%)	Total (%)
**Number of care seekers interviewed**	82	49	131
**Number referred to laboratory**	60 (73.2%)	38 (77.6%)	98 (74.8%)
**Number tested**	60 (100.0%)	36 (94.7%)	96 (98.0%)
**Time spent at laboratory**			
< 1 hour	52 (86.7%)	29 (76.3%)	81 (84.4%)
1-2 hours	3 (5.0%)	1 (2.6%)	4 (4.1%)
Don’t know	5 (8.3%)	6 (16.7%)	11 (11.5%)
**Reported results to HW**	54 (90.0%)	34 (89.5%)	88 (89.8%)
**HW explained results to care seeker**	40 (74.1%)	27 (79.4%)	67 (76.1%)

#### Household survey

##### Knowledge of malaria symptoms, causes, risks and treatment among women of reproductive age

Of the 551 women aged 15 to 49 years interviewed regarding knowledge of malaria symptoms, causes, prevention and treatment. Fever as malaria symptom was mentioned by 86.1% (Kano: 88.1%, Zamfara: 84.9%) and 90% cited mosquitoes as the cause of malaria. Children were mentioned as most vulnerable to malaria by 63.0% (Kano 63.6% and Zamfara 63.0%). A larger proportion of women in Kano (30.2%) than Zamfara (15.9%) mentioned ACT as treatment for malaria. Among women with tertiary education, a larger proportion (64%) mentioned ACT as the treatment for malaria, compared to (12.1%) women with only Quranic education (95% CI 31.61 - 68.16). Of women with live birth in the past 2 years, IPT1, IPTp2 and IPTp3 coverages were 38.6%, 37.1% and 24.2% in Kano State and 33.3%, 25.0% and 41.7% in Zamfara respectively (Table 6).

**Table 6:** knowledge of medicine used for malaria treatment among women interviewed who said malaria can be treated

			Drugs						
Characteristics	Number of women who have heard of malaria (N =520)	Women who said malaria can be treated N = 489 n (%)	ACT	Quinine	Artesunate	Chloroquine	Fansidar (SP)	Aspirin/ Paracetamol	Other
**State**									
Kano	236	225 (95.3)	68 (30.2)	0 (0.0)	8 (3.6)	12 (5.3)	38 (16.9)	14 (6.2)	66 (29.3)
Zamfara	284	264 (93.0)	42 (15.9)	6 (2.3)	17 (6.4)	19 (7.2)	30 (11.4)	13 (4.9)	58 (22.0)
**Education**									
Primary	56	54 (96.4)	12 (22.2)	0 (0.0)	2 (3.7)	0 (0.0)	10 (18.5)	3 (5.6)	0 (0.0)
Secondary	90	88 (97.8)	32 (36.4)	2 (2.3)	8 (9.1)	12 (13.6)	22 (25.0)	7 (8.0)	33 (37.5)
Tertiary	26	25 (96.2)	16 (64.0)	1 (4.0)	3 (12.0)	3 (12.0)	5 (20.0)	1 (4.0)	7 (28.0)
Quranic	184	174 (94.6)	21 (12.1)	1 (0.6)	4 (2.3)	8 (4.6)	16 (9.2)	12 (6.9)	51 (29.3)
No education	164	148 (90.2)	29 (19.6)	2 (1.4)	8 (5.4)	8 (5.4)	15(10.1)	4 (2.7)	19 (12.8)

##### Ownership and use of nets

Surveyed heads of household reported that household ownership of at least one LLIN was 62.3% in Kano and 62.9% in Zamfara but those with one LLIN per 2 persons was 27.1% Kano and 30% in Zamfara. The universal coverage of LLIN is lower than the national target of 58% in 20165. Over 70% of nets were received through a mass campaign conducted in 2015. Other sources of LLIN acquisition included ANC clinic visits (Zamfara: 6.2%, Kano: 5.0%) and immunization clinics (Zamfara: 3.6%, Kano: 1.1%). LLIN use the night before the survey among households who owned LLIN was 67.9% in Zamfara and 68.0% in Kano. LLIN use was higher among women (Kano: 73.2%, Zamfara: 81.0%) and among pregnant women (Kano: 70.8%, Zamfara: 81.6%). Reasons for non-use of LLINs included nets being too old, LLIN gives heat, LLIN causes claustrophobia, LLIN being difficult to hang, LLIN causes itching and has smell (Table 7).

**Table 7:** household LLIN ownership and use

	Characteristics	Kano n (%)	Zamfara n (%)
**Total HHs surveyed** (Kano: 207; Zamfara: 210)	**LLIN ownership** HHs with at least one net	129 (62.3)	132 (62.9)
**Number of persons who slept on the night before the survey in households with at least one net** (Kano: 990; Zamfara: 1103)	**LLIN use** Number of persons who slept inside the net the night before the survey	673 (68.0)	749 (67.9)
**Total number of LLIN sources** (Kano: 281; Zamfara: 276)	**Sources of LLIN**		
	Mass campaign	220 (78.3)	219 (79.3)
	ANC clinics	14 (5.0)	17 (6.2)
	Immunization clinics	3 (1.1)	10 (3.6)
	Others	44 (15.7)	30 (10.9)
**Total number of women** (Kano: 235; Zamfara: 279)	**LLIN use** Women who slept inside LLIN on the night before survey	172 (73.2)	226 (81.0)
**Total number of pregnant women** (Kano: 24; Zamfara: 38)	**LLIN use** Pregnant women who slept inside LLIN on the night before survey	17 (70.8)	31 (81.6)
**Among women with live births in the last 2 years preceding the survey**			
	IPTp1	38.6%	33.3%
	IPTp2	37.1%	25.0%
	IPTp3	24.2%	41.7%

## Discussion

The assessment found that most HW were knowledgeable of the national malaria guidelines especially diagnosis and treatment of uncomplicated malaria. The national guidelines in 2016 recommend quinine for treatment of uncomplicated malaria in first trimester but over a third of HWs said they would use SP or ACT to treat malaria illness in pregnant women in their first trimester. Our study revealed high awareness of cause, symptoms, at-risk groups, and prevention practices for malaria among women 15-49 years of age. However, there was low awareness among women interviewed on use of ACT for malaria treatment.

The proportion of LLIN use among pregnant women in Kano: 70.8% and Zamfara: 81.8 % is lower than the national target for 2016. Mass campaigns are the main source of LLINs for households in both States. Kano and Zamfara States are due for net replacement campaigns in 2019; however, it is doubtful if this strategy is sustainable as it is donor dependent. LLIN use and IRS have estimated combined protective efficacy of 55% for reducing malaria-attributable mortality among children 1-59 months and IPTp and LLIN use have an expected 35% efficacy for reducing low birth weight in sub-Saharan Africa [9,10]. There is therefore an urgent need to strengthen other routes of LLIN acquisition.

Key informants in both States mentioned regular use of RDTs for malaria confirmation. This would reduce presumptive treatment of all fever cases as malaria and therefore generate higher quality data for malaria surveillance. Unfortunately, this was not the observation from HW practice through exit client interview.

Based on HW responses, ACTs were the most common medicines administered for malaria treatment. This may be due to the acceptance of the national policy on malaria treatment, which recommends ACT as the drug of choice, and the availability of job aids on the new national malaria guidelines and capacity building of health workers. However, a small proportion of health workers still prescribed non-recommended medicines, such as chloroquine and ACT or SP in the first trimester for pregnant women, contrary to national guidelines.

### Limitations

During the data review different versions of registers were in use in health facilities (2010 and 2013 versions). The sampling strategy and statistical analysis used in the health facility and community surveys are not intended to produce representative population-based estimates. Our methods were meant to provide practical and actionable data for local health planners and managers and should not be interpreted in the same way as estimates from systematic national or statewide surveys like Malaria Indicator Survey (MIS) or Demographic Health Survey (DHS).

## Conclusion

We recommend continuous orientation on the national guidelines and supportive supervision from higher levels to ensure adherence to the guidelines. There should be frequent problem-solving trainings for HW. Alternate sources should be explored to increase access to LLIN for the community. Prevention of malaria in pregnancy is important and need strategy to increase IPTp coverage. Non-recommended antimalarials like chloroquine should be withdrawn from circulation in public facilities.

### What is known about this topic

- Malaria control has improved with use of efficacious interventions;
- Bed net ownership and use have increased.

### What this study adds

- In Kano and Zamfara States, Nigeria; gap exit between clinicians´ knowledge of the national malaria treatment guidelines and adherence to same;
- Malaria prevention practices are sub-optimal compared to the national targets.
